# Multi-Trait Genomic Prediction of Meat Yield in Pacific Whiteleg Shrimp (*Penaeus vannamei*)

**DOI:** 10.3390/ani15081165

**Published:** 2025-04-18

**Authors:** Shiwei Zhang, Jie Kong, Jian Tan, Xianhong Meng, Ping Dai, Jiawang Cao, Kun Luo, Mianyu Liu, Qun Xing, Yi Tian, Juan Sui, Sheng Luan

**Affiliations:** 1 College of Fisheries and Life Science, Dalian Ocean University, Dalian 116023, China; zsw13831811936@163.com (S.Z.); tianyi@dlou.edu.cn (Y.T.); 2 State Key Laboratory of Mariculture Biobreeding and Sustainable Goods, Yellow Sea Fisheries Research Institute, Chinese Academy of Fishery Science, Qingdao 266071, China; kongjie@ysfri.ac.cn (J.K.); tanjian@163.com (J.T.); mengxianhong@ysfri.ac.cn (X.M.); daiping54@163.com (P.D.); caojw@ysfri.ac.cn (J.C.); luokun@ysfri.ac.cn (K.L.); 2022213007@stu.njau.edu.cn (M.L.); 3 Laboratory for Marine Fisheries Science and Food Production Processes, Qingdao Marine Science and Technology Center, Qingdao 266237, China; 4 College of Fisheries, Nanjing Agricultural University, Nanjing 210095, China; 5 BLUP Aquabreed Co., Ltd., Weifang 261311, China; xingqun527@163.com

**Keywords:** *Penaeus vannamei*, meat yield, net meat weight, heritability, multi-trait genomic prediction

## Abstract

The meat yield (MY) is a key economic trait in Pacific whiteleg shrimp (*Penaeus vannamei*) breeding, necessitating accurate genomic prediction for efficient genetic improvement. In this study, we investigated single-trait (STGMs) and multi-trait genomic models (MTGMs) for predicting MY and related traits, using two cross-validation strategies reflecting different data-availability scenarios. A total of 899 individuals from 63 full-sibling families were phenotyped for MY, net meat weight (MW), body weight (BW), body length (BL), and abdominal segment length (AL). We estimated the genomic heritability and genetic correlations of MY and related traits in *P. vannamei*, followed by comparing the prediction accuracy of STGMs and MTGMs for MY and MW. Two validation approaches were then applied: CV1 retained auxiliary traits in the validation sets, and CV2 excluded both target and auxiliary traits. Heritability estimates indicated that MY had low heritability (STGM: 0.160; MTGMs: 0.145–0.156), whereas MW, BW, BL, and AL showed low-to-moderate heritability (0.099–0.204). Genetic correlations revealed strong associations between MY and MW/BW/BL (*r_g_* = 0.605–0.783), yet a low positive correlation with AL (*r_g_* = 0.286). Across all comparisons, MTGMs consistently surpassed STGMs. For MY, MTGMs improved the accuracy by 4.8–58.8% relative to STGM (0.187), with the MY-MW model achieving the highest accuracy (0.297) under CV1. Similarly, MTGMs enhanced MW prediction by 36.6–138.2% over STGM (0.254), with the MW-BW model reaching the highest accuracy (0.605) under CV1. Notably, retaining auxiliary traits (CV1) boosted accuracy gains substantially (up to 138.2%), whereas excluding them (CV2) yielded only marginal improvements (≤8.6%). Moreover, incorporating AL as an auxiliary trait increased heritability estimates for MW, BW, and BL by 5.4–7.6%, indicating its synergistic value in MTGMs. Overall, these results demonstrate that MTGMs markedly enhance genomic prediction for carcass traits compared to STGMs, particularly when auxiliary trait data are accessible (CV1). The findings underscore the importance of maintaining auxiliary trait records in breeding populations, offering a robust framework for improving *P. vannamei* through multi-trait genomic prediction models.

## Simple Summary

Enhancing the meat yield in Pacific whiteleg shrimp (*Penaeus vannamei*) is vital for aquaculture profitability. In this study, we compared single-trait and multi-trait genomic models using 899 shrimp from 63 families to predict meat yields and related traits. Results showed that multi-trait genomic models were superior to single-trait ones. When auxiliary traits (like net meat weight) were added to the validation set to build multi-trait genomic models, the prediction accuracy of the meat yield could increase by 58.8%. But if auxiliary traits were not in the validation set, such models only slightly boosted the meat yield prediction accuracy by up to 8.6%. These findings underscore the value of multi-trait genomic models in shrimp breeding programs, particularly when supplementary trait data are accessible, and highlight a practical route to accelerate improvements in commercial aquaculture.

## 1. Introduction

The Pacific white shrimp (*Penaeus vannamei*) is currently the most widely farmed aquaculture species worldwide [1]. Introduced to China in the late 1980s, it has attracted significant attention due to its rapid growth, strong stress resistance, and high nutritional value. Consequently, its production in China has continuously expanded, reaching 2.24 million tons in 2023 [2]. Among the many factors influencing the economic efficiency of shrimp farming, body weight (BW) is a primary economic trait and has been the target in selective breeding programs [3]. However, because the shrimp cephalothorax has relatively low edible value, the proportion of abdominal muscle becomes especially important. This proportion, commonly referred to as meat yield (MY), is defined as the ratio of abdominal muscle weight to the BW. Increasing the MY can significantly enhance the market value and quality of shrimp products [4]. Therefore, the MY has gradually become an important breeding criterion [5].

The Pedigree-based Best Linear Unbiased Prediction (pBLUP) method has been widely adopted in aquaculture for predicting the estimated breeding value (EBV), due to its capability to account for shared family relationships [6]. Nevertheless, for traits, such as MY, that typically require sacrificing individuals for measuring, pBLUP-based selection becomes constrained. Moreover, in sib-selection scenarios, pBLUP primarily exploits family-level information and may overlook genetic variation among individuals within the same family, thereby reducing the accuracy of the genetic evaluation [3,7].

By contrast, genomic selection can mitigate Mendelian sampling errors and produce more accurate estimates of the individual genomic EBV (GEBV) [8]. Among these approaches, Genomic Best Linear Unbiased Prediction (GBLUP) [9] has shown notable improvements in aquaculture traits, including both disease resistance and the meat yield. For instance, in *Fenneropenaeus merguiensis*, GBLUP increased the prediction accuracy by 567% for Hepatopancreatic parvovirus resistance and 16.92% for the net meat weight (MW), respectively, relative to pBLUP [10]. Likewise, in finfish, GBLUP-based evaluations have yielded promising results for carcass traits. In rainbow trout (*Oncorhynchus mykiss*), the MY accuracy rose by 35%, and in channel catfish*,* the MW accuracy improved by 36%, both in comparison with pBLUP [11,12]. Despite these advances, genomic evaluations of the MY in shrimp remain relatively sparse and have thus far centered mainly on *F. merguiensis* [13], with limited research on *P. vannamei*.

Most current evaluations of the MY still employ single-trait animal models, which directly assess the genetic potential of the MY and select individuals with higher breeding values. However, single-trait models do not harness the genetic correlations among different traits. In contrast, multi-trait animal models can simultaneously incorporate information from multiple traits, thereby improving the accuracy of genetic evaluations [14,15]. For instance, Song et al. (2022) [16] simulated growth traits with a heritability of 0.3 and disease resistance traits with a heritability of 0.1 in rainbow trout. When the genetic correlation between the two traits was 0.5, the multi-trait model improved the prediction accuracy by 0.002 for growth traits and by 0.064 for disease-resistance traits compared to the single-trait model [16]. In *Lates calcarifer*, retaining other correlated body size traits in the validation set for a multi-trait model increased the MY prediction accuracy by 5% to 76% compared with excluding those traits [17].

In *P. vannamei*, the genetic evaluations of MY thus far has have largely relied on pBLUP [18], and there are no known studies that combine genomic data with multi-trait models for improving the MY. Therefore, in this study, we used genomic information to evaluate the MY trait in *P. vannamei,* comparing the performances of single-trait and two-trait animal models. Our results demonstrate that the multi-trait model outperforms single-trait models in different validation schemes, offering a valuable strategy to enhance the MY in commercial shrimp breeding.

## 2. Materials and Methods

### 2.1. Data Collection

#### 2.1.1. Experimental Materials

The experimental shrimp were sourced from a nucleus breeding population maintained by BLUP *Aquabreed* Co., Ltd. (Weifang, Shandong, China). A total of 63 full-sibling families were established, with a 5-day age gap between the oldest and youngest families. During the juvenile stage, each family was reared separately under standardized water-quality-control, feeding, and daily-management protocols [19]. Once shrimp from all 63 full-sibling families reached 40–45 days post-hatching under standardized rearing conditions, 20 healthy individuals were randomly selected from each family, yielding a total of 1260 individuals. Juvenile shrimp from 10–11 families were co-cultured in each 3 m^2^ concrete tank, and six tanks in total were used for growth performance testing. Due to the layout of the experimental area, three tanks were exposed to high light intensity (light intensity: 267.78 ± 40.61 lx), while the other three experienced low light intensity (light intensity: 48.85 ± 6.15 lx). Consequently, the six tanks were classified into two groups (A and B) based on differences in light exposure. Other rearing conditions, including the feeding regime and water parameters, were kept consistent across all tanks.

#### 2.1.2. Growth Traits Test and Measurement

After the growth test, all shrimp were slaughtered at 174–178 days of age. Group A had an average survival rate of 83.29%, while Group B had an average survival rate of 76.94%. The *t*-test showed an extremely significant difference (*p* < 0.01) between the two groups (Table 1). A total of five traits were analyzed, including three growth-related traits and two carcass traits. The growth traits included the BW, body length (BL), and abdominal segment length (AL), which were measured using an computer-assisted measurement system. Harvested shrimp were first temporarily placed in pre-cooled water (below 18 °C) in a plastic bucket with aeration to reduce activity. They were then removed, their surface water was absorbed using absorbent paper, and they were placed into the system’s image acquisition box for lateral image capture, where the BW, BL, and AL were recorded. Specifically, BL is measured from the base of the eye stalk to the distal end of the telson, while AL spans from the first to the sixth abdominal segments. The sex of each shrimp was manually recorded. The carcass traits included the MW and MY. The MW was measured by manually separating the entire abdominal meat and weighing it on an electronic balance with a precision of 0.01 g. The MY for each shrimp was calculated using the following formula: MY = MW/BW × 100%. After excluding a small number of abnormal data points, 899 shrimp were retained for further analysis. Statistical analyses, including basic descriptive statistics, such as the mean, maximum, minimum, standard deviation, and coefficient of variation, were generated using Microsoft Excel 2016.

### 2.2. Genotyping

After morphometric measurements, muscle tissue samples were collected from each individual and stored in 3 mL of DNA preservation solution (*Molbreeding Biotechnology Company, Shijiazhuang, China* (https://www.molbreeding.com, accessed on 10 May 2023)). Genomic DNA was extracted from these samples for subsequent genotyping. Genotyping was conducted using the liquid-phase chip “Yellow Sea Array No. 1”, specifically utilizing its 10K SNP panel. Initially, 10,291 SNPs were retained. The genotyping data underwent quality control using PLINK (v1.90) [20], applying filtering criteria that included an individual genotype call rate exceeding 80%, an SNP call rate above 90%, and a minor allele frequency (MAF) greater than 5%. After filtering, the SNP call rate was 97.68%, and 9227 high-quality SNPs were retained for further analysis.

### 2.3. Genetic Parameter Estimation

The genomic relationship matrix (G-matrix) was constructed using the preGSf90 program in BLUPF90 1.70 [21,22], according to the following formula:

$$G=\frac{ZZ’}{2\sum p_{i}(1-p_{i})},$$

where *Z* is an *n* × *m* matrix of centered SNP genotypes (*n* = number of genotyped individuals, *m* = number of SNPs), and *p_i_* is the alternative allele frequency for the *i*th SNP.

Variance components of the MY and other related traits were estimated using the Average Information Restricted Maximum Likelihood (AIREML) method, using the G-matrix and an animal model implemented in ASReml-R V4.1.0 [23,24].

#### 2.3.1. Single-Trait Genomic Model (STGM)

The general form of the STGM was expressed as follows:

y=Xb+Zu+e

where *y* is the vector of phenotypes (e.g., MY, MW, BW, BL, and AL), *b* is the vector of fixed effects (including gender, tank group, and the interaction between tank group and gender), *u* is the vector of random individual additive genetic effects, *e* is the random residual vector, and *X* and *Z* are the corresponding incidence matrices for fixed and random effects, respectively.

#### 2.3.2. Multi-Trait Genomic Models (MTGMs)

The general form of the MTGM was expressed as follows:

$$\left[\begin{array}{l} y_{1}\\ y_{2} \end{array}\right]=\left[\begin{array}{l} X_{1}\;0\\ 0\;X_{2} \end{array}\right]\left[\begin{array}{l} b_{1}\\ b_{2} \end{array}\right]+\left[\begin{array}{l} Z_{1}\;0\\ 0\;Z_{2} \end{array}\right]\left[\begin{array}{l} u_{1}\\ u_{2} \end{array}\right]+\left[\begin{array}{l} e_{1}\\ e_{2} \end{array}\right],$$

where *y*_1_ and *y*_2_ are the phenotype vectors of the two traits to be analyzed, *b*_1_ and *b*_2_ are the fixed effect vectors for the two traits (including gender, cement tank group, and the interaction between cement tank group and gender), *u*_1_ and *u*_2_ are the random individual additive genetic effects vectors for the two traits, *e*_1_ and *e*_2_ are the random residual vectors for the two traits, *X*_1_ and *X*_2_ are the association matrices for the fixed effects of the two traits, and *Z*_1_ and *Z*_2_ represent the association matrices for the random effects of the two traits.

#### 2.3.3. Heritability and Genetic Correlations

Heritability (*h*^2^) for the MY and other traits was estimated based on variance components derived from the STGM and MTGM. The heritability was calculated as follows:

(1) $$h^{2}=\sigma_{a}^{2}/(\sigma_{a}^{2}+\sigma_{e}^{2}),$$

where $\sigma_{a}^{2}$ is the additive genetic variance and $\sigma_{e}^{2}$ is the residual variance.

Genetic correlations ($r_{g}$) between traits was derived from the MTGM, using the follow formula:

(2) $$r_{g}={cov}_{g}(x,y)/\sigma_{g}(x)\cdot \sigma_{g}(y),$$

where ${cov}_{g}(x,y)$ is the genetic covariance component between traits *x* and *y*, and $\sigma_{g}(x)$ and $\sigma_{g}(y)$ are the square roots of the genetic variances for each trait, respectively.

To test whether estimates of heritability or genetic correlation significantly differed from theoretical values (e.g., 0 or 1), a Z-score [25] was used. Significance thresholds were set at |*Z*| ≥ 1.96 (*p* < 0.05) and |*Z*|≥ 2.58 (*p* < 0.01)

### 2.4. Evaluation of Genomic Prediction Accuracy

The STGM and MTGM were used to predict GEBVs of carcass traits (MY and MW) in *P. vannamei*. A 5-fold cross-validation scheme was implemented to evaluate the prediction accuracy and bias of the GEBV. All genotyped individuals were randomly divided into five equal subsets, with one subset designated as the validation set. In the STGM approach, the phenotypic data for the single target trait were masked in the validation set. For the MTGM, two validation strategies were applied: in CV1, only the target trait data were masked while retaining the phenotypic data of auxiliary traits, whereas in CV2, both the target trait and auxiliary trait data were masked, completely excluding auxiliary trait information. The remaining four subsets were used as the training set for GEBV prediction in the validation set. The average Pearson’s correlation coefficient between the observed phenotypes and the predicted GEBVs in the validation set was used as a measure of predictive accuracy. The slope of the regression of observed phenotypes based on GEBVs was used to evaluate predictive bias. A bias of 1 indicates that the GEBV is an unbiased estimate of the true breeding value. A regression coefficient equal to 1 indicates an unbiased prediction, whereas values above or below 1 suggest underestimation or overestimation, respectively. The validation for each trait was repeated 5 times, and the average value was taken as the evaluation metric for model accuracy.

## 3. Results

### 3.1. Descriptive Statistics of Growth Traits

Descriptive statistics for the MY, MW, BW, BL, and AL in the 899 sampled shrimp are presented in Table 2. The mean MY was 50.56%, with a coefficient of variation (CV) of 4.91%. In comparison, the MW and BW had mean values of 13.81 g and 27.29 g, respectively, with CVs of 17.44% and 16.31%, both higher than that of MY. Meanwhile, the BL and AL averaged 13.04 cm and 7.50 cm, with CVs of 5.73% and 6.22%, respectively, which are similar to that of MY.

### 3.2. Heritability and Genetic Correlation

The heritability estimates for the MY, MW, BW, BL, and AL based on STGMs and MTGMs are summarized in Table 3. The STGM estimated low to medium heritability for the MY (0.160), MW (0.197), and growth traits (BW: 0.158, BL: 0.133, AL: 0.101). The MTGM generally produced lower MY heritability estimates (0.145–0.156) compared to the STGM (0.160), but incorporating AL as an auxiliary trait slightly increased the MY heritability (h^2^ = 0.156, a 5.4–7.6% gain). The MW heritability ranged from 0.195 to 0.204, while the BW, BL, and AL showed heritability estimates between 0.099 and 0.167. Notably, MTGMs incorporating AL as an auxiliary trait yielded higher heritability estimates for the MW, BW, and BL than STGMs. All heritability estimates were significantly different from zero (*p* < 0.01), suggesting that genetic variation plays a role in these traits though the genetic contribution remains relatively low.

Using the MTGM, genetic correlations among the MY, MW, BW, BL and AL are shown in Table 4. The MY had medium positive correlations with the MW, BW, and BL (0.605–0.783), but the correlation between the MY and AL was relatively low (0.286), indicating a mildly positive association. The genetic correlations among the MW, BW, BL, and AL ranged from 0.735 to 0.978, all indicating strong positive relationships.

### 3.3. Genomic Prediction

The prediction accuracy and bias for the MY were evaluated using STGM and MTGMs (Table 5). With the STGM, the accuracy of the MY GEBV prediction was 0.187. In contrast, MTGMs demonstrated substantial improvements under the CV1 strategy, with prediction accuracies ranging from 0.196 to 0.297, representing improvements of 4.8–58.8% compared to the STGM. Among the MTGMs (MW–MY, MW–BW, MW–BL, and MW–AL), the MY–MW model yielded the highest accuracy (0.297), demonstrating optimal performance under CV1. Under CV2, the improvements were more modest, with accuracies ranging from 0.189 to 0.203 (1.07–8.56% gain), and the MY–MW model again performed best (0.203).

Table 6 summarizes the prediction accuracy and bias for the MW using the STGM and MTGMs. The STGM approach yielded an accuracy of 0.254, while MTGMs, particularly under CV1, substantially improved the predictive performance. The MW–BW model achieved the highest accuracy (0.605), representing a 138.19% improvement over the STGM. Across the four MTGMs, CV1 accuracies ranged from 0.466 to 0.605, reflecting enhancements of 36.61 to 138.19% relative to the STGM. In contrast, under CV2, the accuracy range was 0.252 to 0.265, with marginal improvements of 0.79 to 4.33%, while MW–AL showed a slight decline of −0.79% compared to the STGM.

## 4. Discussion

Enhancing the MY is a key objective in shrimp breeding due to its direct impact on profitability and product quality. In this study, we compared STGMs and MTGMs for predicting the MY and related traits. This is the first study to evaluate the MY using multi-trait GBLUP in *P. vannamei*. Our results provide valuable insights into the genetic architecture of the MY and highlight the advantages of incorporating auxiliary traits in genomic selection strategies.

### 4.1. Heritability Estimates and Influencing Factors

A key finding was the relatively low heritability of the MY, estimated between 0.145 and 0.160, which is lower than the 0.231 reported by Dai et al. (2023) [18]. This discrepancy may stem from differences in shrimp populations, growth stages, or rearing conditions. Our population was older (~200 days) and heavier (mean BW ~27.29 g) than that in Dai’s study (~60 days post-growth to 5 cm, mean BW ~11.87 g), potentially reducing the proportion of genetic variance attributable to the MY. Previous studies indicate that heritability estimates can vary across developmental stages [26,27,28,29]. For instance, Li et al. (2017) [26] reported that BW heritability in *Paralichthys olivaceus* declined from 0.35 at 180 days to 0.13 at 360 days, with similar trends observed in *Takifugu rubripes* [27], *Portunus trituberculatus* [28], and *Exopalaemon carinicauda* [29]. Furthermore, although common environmental effects were not explicitly modeled, we minimized the potential bias by employing early commingling (P40–P45) and standardized rearing over 174–178 days, well beyond the initial family-specific phase. Although few studies have estimated MY heritability in shrimp using genomic methods, a GBLUP-based estimate of 0.10 was reported in *F. merguiensis* [30]. In *Oreochromis niloticus* [31,32], heritability estimates for the MY ranged from 0.17 when common environmental effects were ignored to 0.210 when they were accounted for using ssGBLUP. Collectively, these findings suggest that MY heritability is influenced by growth stage, rearing conditions, and modeling approaches.

### 4.2. Genetic Correlations and Multi-Trait Genomic Predictions

Genetic correlations between the MY and related traits were consistently strong, with values of 0.783 for the MW, 0.636 for the BW, and 0.605 for the BL. These estimates align with previous studies on *P. vannamei* [18,33] and are comparable to those reported in other aquaculture species. In *O. niloticus*, Rutten et al. (2005) [34] found genetic correlations of 0.74 (MY–BW) and 0.62 (MY–BL), while Nguyen et al. (2010) [35] estimated MY–BW and MY–BL correlations of 0.44 ± 0.20 and 0.38 ± 0.18, respectively.

MTGMs, which explicitly leverage these genetic correlations [36], demonstrated superior predictive accuracy over STGMs. Notably, the MY–MW model achieved the highest improvement in MY prediction, increasing the accuracy by 58.8% compared to STGMs. Additionally, the MY–BW model improved the MY prediction from 0.187 to 0.204, while for the MW, the MW–BW and MW–BL models increased the prediction accuracy by 138.2% and 119.3%, respectively, over STGMs. The MW–BW combination achieved the highest accuracy (0.605) but also exhibited the largest bias (2.093). The bias arises from the CV1 retaining BW in the validation set. The strong genetic correlation (*r_g_*= 0.978) led to overreliance on BW, compressing GEBV variation for MW and inflating bias. Increasing the number of tested individuals may improve this, but further experimental validation is needed. These findings underscore the advantages of MTGMs in carcass trait prediction.

### 4.3. Impact of Validation Strategies on Prediction Accuracy

To assess the impact of auxiliary traits on genomic prediction, we compared two validation strategies: CV1, where auxiliary traits were retained in the validation set, and CV2, where both target and auxiliary traits were excluded. Retaining auxiliary traits (CV1) significantly boosted the prediction accuracy (up to 138.2%), whereas excluding them (CV2) resulted in only marginal gains (≤8.6%). These results underscore the critical importance of maintaining comprehensive auxiliary trait records in breeding programs. Consistent with our findings, a study on *L. calcarifer* demonstrated that including correlated body size traits in the validation set led to substantial improvements in MY prediction accuracy, with gains ranging from 5% to 76% compared to multi-trait GBLUP [17]. Similarly, in plant breeding, Kristensen et al. (2019) [37] reported that incorporating auxiliary traits, such as a falling number and Zeleny sedimentation value in multi-trait models, improved the predictive ability for the grain protein content, increasing accuracy from 0.13 to 0.17. This improvement was also attributed to retaining auxiliary traits in the validation set, as applied in CV1.

Given the high accuracy of the MTGM in trait evaluations, it is suggested that in practical breeding, the target trait and the trait with a high genetic correlation can be included in the MTGM model to enhance the evaluation accuracy. If both traits can still participate in subsequent breeding after testing, the CV1 scheme can be used, and if the tested individuals cannot be used for breeding, the CV2 scheme is suggested.

## 5. Conclusions

This study assessed the effectiveness of the STGM and MTGM for predicting the MY in *P. vannamei.* The heritability of the MY was low (0.145–0.160), likely influenced by the growth stage and rearing conditions. Strong genetic correlations between the MY and MW, BW, and BL supported the use of MTGMs. MTGMs outperformed STGMs, with the MY–MW model improving MY prediction by 58.8%, while MW prediction was enhanced by 138.2% and 119.3% using MW–BW and MW–BL models, respectively. Retaining auxiliary traits in the validation set significantly boosted accuracy, underscoring the value of multi-trait records in breeding. These findings highlight the potential of MTGM for genomic selection in shrimp breeding.

## Figures and Tables

**Table 1:** Survival status of *P. vannamei* in six concrete tanks.

Group	Concrete Tanks	Number of Families	Number of Stocking Individuals	Number of Harvested Individuals	Survival Rate (%)
A	A1	10	200	167	83.50 ^a^
	A2	11	220	183	83.18 ^a^
	A3	11	220	183	83.18 ^a^
B	B1	10	200	157	78.50 ^b^
	B2	11	220	169	76.82 ^b^
	B3	10	200	151	75.50 ^b^
Summary	-	63	1260	1010	80.16

Notes: different letters (^a, b^) indicate extremely significant differences (*p <* 0.01).

**Table 2:** Descriptive statistics of the meat yield (MY), net meat weight (MW), body weight (BW), body length (BL), and abdominal segment length (AL) in *P. vannamei*.

Trait	Mean	Max	Min	SD	CV (%)
MY (%)	50.56	61.11	40.97	2.48	4.91
MW (g)	13.81	23.36	5.58	2.41	17.44
BW (g)	27.29	44.20	12.00	4.45	16.31
BL (cm)	13.04	15.26	10.00	0.75	5.73
AL (cm)	7.50	8.81	4.91	0.47	6.22

Notes: Max, maximum; Min, minimum; SD, standard deviation; CV, coefficient of variation.

**Table 3:** Heritability estimates for the meat yield (MY), net meat weight (MW), body weight (BW), body length (BL), and abdominal segment length (AL) in *P. vannamei* based on the single-trait genomic models (STMGs) and multi-trait genomic models (MTGMs).

Trait	MY	MW	BW	BL	AL
MY	**0.160 ± 0.048 ^a^**	0.197 ± 0.049 ^a^	0.152 ± 0.045 ^b^	0.130 ± 0.043 ^b^	0.101 ± 0.039 ^a^
MW	0.145 ± 0.046 ^b^	**0.197 ± 0.050 ^a^**	0.152 ± 0.045 ^b^	0.126 ± 0.042 ^b^	0.099 ± 0.038 ^b^
BW	0.145 ± 0.046 ^b^	0.197 ± 0.049 ^a^	**0.158 ± 0.046 ^a^**	0.130 ± 0.043 ^b^	0.099 ± 0.038 ^b^
BL	0.148 ± 0.047 ^b^	0.195 ± 0.048 ^a^	0.158 ± 0.046 ^a^	**0.133 ± 0.044 ^a^**	0.101 ± 0.038 ^a^
AL	0.156 ± 0.048 ^a^	0.204 ± 0.049 ^b^	0.167 ± 0.047 ^b^	0.140 ± 0.045 ^b^	**0.101 ± 0.039 ^a^**

Notes: “^a^” indicates non-significant difference between MTGM and STGM estimates, while “^b^” indicates a significant difference. The diagonal values represent the heritability of the corresponding traits estimated using single-trait genomic models. The off-diagonal elements represent the heritability estimates of the target trait, constructed using the trait in the bolded column name as the target trait and the trait in the non-bolded row name as the auxiliary trait in a multi-trait genomic model. For example, the value 0.145 ± 0.046 represents the heritability estimate for the meat yield (MY), constructed using the multi-trait genomic model that includes the MY and either net meat weight (MW) or body weight (BW) as traits.

**Table 4:** Genetic correlations between the meat yield (MY), net meat weight (MW), body weight (BW), body length (BL), and abdominal segment length (AL) of *P. vannamei* using the multi-trait genomic models.

Trait	MY	MW	BW	BL	AL
MY	1	-	-	-	-
MW	0.783 (0.133)	1	-	-	-
BW	0.636 (0.200)	0.978 (0.013)	1	-	-
BL	0.605 (0.213)	0.915 (0.040) ^b^	0.935 (0.029) ^b^	1	-
AL	0.286 (0.248)	0.735 (0.106) ^b^	0.811 (0.089) ^b^	0.924 (0.047)	1

Notes: the values below the diagonal represent the genetic correlation coefficients between traits based on the multi-trait genomic models. “^b^” indicates a significant difference from 1 in the statistical test (*p* < 0.05).

**Table 5:** The prediction accuracy and bias of breeding values for the meat yield using the single-trait genomic model (STMG) and multi-trait genomic models (MTGMs).

Model	Validation Strategies	Trait	Accuracy	Bias
STGM	CV2	MY	0.187	0.972
MTGM	CV1	MY–MW	0.297	1.326
		MY–BW	0.204	1.018
		MY–BL	0.197	0.973
		MY–AL	0.196	1.031
	CV2	MY–MW	0.203	1.012
		MY–BW	0.199	1.002
		MY–BL	0.195	0.985
		MY–AL	0.189	1.046

Notes: MY, meat yield; MW, net meat weight; BW, body weight; BL, body length; AL, abdominal segment length; CV1, retaining auxiliary traits in validation sets; CV2, excluding both target and auxiliary traits. Trait1–Trait2 indicates that auxiliary trait 2 is retained to construct the MTGM for Trait1 and Trait2, and the prediction accuracy of the Genomic Estimated Breeding Value (GEBV) for Trait1 is evaluated. For example, MY–MW indicates that the MW is retained to construct the MTGM for the MY and MW, and the prediction accuracy of the GEBV for the MY is evaluated.

**Table 6:** The prediction accuracy and bias of breeding values for the net meat weight using the single-trait genomic models (STMGs) and multi-trait genomic models (MTGMs).

Model	Validation Strategies	Trait	Accuracy	Bias
STGM	CV2	MW	0.254	1.021
MTGM	CV1	MW–MY	0.347	1.268
		MW–BW	0.605	2.093
		MW–BL	0.557	1.983
		MW–AL	0.466	1.710
	CV2	MW–MY	0.256	1.006
		MW–BW	0.261	1.032
		MW–BL	0.265	1.046
		MW–AL	0.252	0.984

Notes: MY, meat yield; MW, net meat weight; BW, body weight; BL, body length; AL, abdominal segment length; CV1, retaining auxiliary traits in validation sets; CV2, excluding both auxiliary traits. Trait1–Trait2 indicates that auxiliary trait 2 is retained to construct MTGM for Trait1 and Trait2, and the prediction accuracy of the Genomic Estimated Breeding Value (GEBV) for Trait1 is evaluated.

## Data Availability

The original contributions presented in the study are included in the article; further inquiries can be directed to the corresponding author.
